# Pornography Use in Adolescents and Its Clinical Implications

**DOI:** 10.3390/jcm9113625

**Published:** 2020-11-11

**Authors:** Josep M. Farré, Angel L. Montejo, Miquel Agulló, Roser Granero, Carlos Chiclana Actis, Alejandro Villena, Eudald Maideu, Marta Sánchez, Fernando Fernández-Aranda, Susana Jiménez-Murcia, Gemma Mestre-Bach

**Affiliations:** 1 Department of Psychiatry Psychology and Psychosomatics, Dexeus University Hospital, 08028 Barcelona, Spain; psico.dex@quironsalud.es (J.M.F.); agullo.miquel@gmail.com (M.A.); 2 Ital Universitario Psychiatry Department, University of Salamanca Nursing School, 37007 Salamanca, Spain; amontejo@usal.es; 3 Departament de Psicobiologia i Metodologia de les Ciències de la Salut, Universitat Autònoma de Barcelona, 08193 Barcelona, Spain; roser.granero@uab.cat; 4 Ciber Fisiopatología Obesidad y Nutrición (CIBERObn), Instituto de Salud Carlos III, 28925 Madrid, Spain; ffernandez@bellvitgehospital.cat (F.F.-A.); sjimenez@bellvitgehospital.cat (S.J.-M.); 5 Facultad de Ciencias de la Salud, Universidad Internacional de La Rioja, 26006 La Rioja, Spain; carloschiclana@doctorcarloschiclana.com; 6 Unidad de Sexología Clínica y Salud Sexual, Consulta Dr. Carlos Chiclana, 28003 Madrid, Spain; alejandrovillena@doctorcarloschiclana.com; 7 Departamento de Psicología, Facultad de Medicina, Universidad CEU-San Pablo, 28925 Madrid, Spain; 8 Hospital de Campdevanol, 17530 Girona, Spain; dr.emaideu@gmail.com; 9 Consorcio de Eduacación de Barcelona, 08010 Barcelona, Spain; msanc21@xtec.cat; 10 Department of Psychiatry, Bellvitge University Hospital-IDIBELL, 08907 Barcelona, Spain; 11 Department of Clinical Sciences, School of Medicine, University of Barcelona, 08007 Barcelona, Spain

**Keywords:** sexuality, adolescents, pornography, porn, gender, risky behaviors

## Abstract

(1) Background: The Differential Susceptibility to Media Effects Model (DSMM) suggests that pornography use effects are conditional and they depend on dispositional, developmental, and social differential susceptibility variables. This framework also highlights that the differential susceptibility variables act as predictors of pornography use and as moderators of the effect of pornography on criterion variables. (2) Methods: By administering a survey to *n* = 1500 adolescents, we tested whether these assumptions were met. (3) Results: Pornography use was related to being male and older, having a bisexual or undefined sexual orientation, higher substance use, being non-Muslim, and reporting sexual interest and the use of the media to obtain sexual information. Structural Equation Modeling (SEM) showed that higher levels in the criterion variables were directly related to pornography use, older age, substance use, and being women. Some mediational links also emerged. Pornography use mediated between the age and criterion variables. Moreover, substance use mediated the association between age and gender with the criterion variables. (4) Conclusions: Our findings support the clinical applicability of the theoretical DSMM framework. Knowing adolescent pornography consumers’ profiles and the impact of pornography on this population would allow for the designing of more effective prevention and regulation proposals.

## 1. Introduction

The presence of sexually explicit materials has increased significantly both in mass media and social media [1,2]. Moreover, with the emergence of the Internet, the use of pornography has become widespread throughout the world [3,4]. In the case of adolescents and young adults, recent rates of pornography use have been reported to be around 43% [5]. This increase in consumption patterns may be partly explained by the “Triple A” theory, which highlights easy access to the Internet, the fact that a large part of the population can afford it, and the anonymity that the Internet guarantees to its consumers [6].

Numerous studies have focused on evaluating the use of pornography in this age group and its association with multiple variables. Some authors have tried to define possible profiles of adolescents and young people who consume pornography. For example, Efrati et al. [7] identified that those adolescents who used pornography were usually boys, low on social intimacy, introverted and neurotic, and more overt narcissists, among other factors. In this line, Brown et al. [8] identified three types of pornography users taking into account variables such as age, pornography acceptance, use, motivations for use and religiosity—porn abstainers, auto-erotic porn users and complex porn users.

The Differential Susceptibility to Media Effects Model (DSMM) was designed by Valkenburg and Peter [9] and focuses on microlevel media effects. This model is based on multiple solid theories such as Social Cognitive Theory [10], the Neoassociationist Model [11], the Selective Exposure Theory [12], and the Media Practice Model [13]. The DSMM is structured around four central propositions: (1) Media effects are conditional and depend on dispositional, developmental, and social differential susceptibility variables. (2) Media effects are indirect and cognitive; emotional and excitative media response states mediate the relationship between media use and media effects. (3) The differential susceptibility variables act as predictors of media use and as moderators of the effect of media use on media response states. (4) Media effects are transactional; they influence media use, media response states, and differential susceptibility variables [9].

On the basis of the DSMM framework, Peter and Valkenburg [14] have published a review including studies that have evaluated pornography use in adolescents. In terms of dispositional predictors of pornography use, demographics, personality traits, norm-related variables, sexual interest, and Internet behavior have been explored [14]. It has been suggested that male adolescents are more exposed to pornography than females, although gender differences are smaller the more liberal their country of origin is [15,16,17]. Moreover, rule-breaking and adolescents who use substances may use pornography more frequently [18,19]; the same goes for adolescents with greater sexual interest [20]. 

Regarding developmental variables, age, pubertal maturation, and sexual experience have been studied in adolescents. There is controversy about whether pornography use increases with age, and existing studies reported conflicting results [15,16,18]. In studying possible trajectories of adolescent pornography use, however, it has been suggested that early puberty may be linked with earlier exposure to pornography and more frequent pornography use later [21]. The same applies to sexual experience, with some authors associating it with more frequent pornography use, while others associated it with a lower frequency [15,20]. Taking social variables into account, poor family functioning, desire for popularity, peer pressure, and victimization online and offline have been related with higher pornography use in adolescents [18,22,23,24]. In this vein, Nieh et al. [21] evaluated the influence of factors such as peer behaviors and parenting style on adolescent pornography use trajectories, finding that parental monitoring protected adolescents from pornography use. Relatedly, Efrati et al. [25] highlighted that the impact of loneliness on the frequency of pornography use may be dependent on individuals’ attachment orientations. In terms of victimization, the possible association between the use of pornography and violence and sexual aggression and coercion, as well as the problematic use of pornography, have been particularly studied [26,27,28,29,30]. 

Finally, with regard to criterion variables, pornography use has been related to more permissive sexual attitudes [31,32,33]. However, evidence for an association between pornography use and risky sexual behaviors, such as unprotected sex, is mixed [34,35].

Therefore, the existing evidence on how these multiple variables interact with each other is contradictory, and to the best of our knowledge, no study has yet evaluated all the variables proposed by the DSMM. Therefore, there is still a lack of systematic data on how the multiple variables of the DSMM model interact with each other. To this end, the present study aimed to assess in an integrated way the nuclear correlates of the use of pornography in adolescents suggested by the DSMM (dispositional, developmental, social and criterion variables). For this purpose, we tested two of the four DSMM propositions: (1) we explored whether dispositional, developmental, and social variables predict pornography use; (2) we evaluated whether dispositional, developmental, and social variables may not only predict pornography use but also moderate the extent to which pornography use predicts criterion variables. We hypothesized that the explored DSMM proposals will be fulfilled.

## 2. Experimental Section

### 2.1. Participants and Procedure

An e-mail was sent to all the public and private high schools in Catalonia (Spain) that appeared on the list provided by the Catalan government. Special education centers were excluded. Of all the high schools, excluding those that did not answer or refused to participate, 14 schools were finally included, with a total of *n* = 1500 adolescent students (14–18 years old). It was the principals or boards of education who gave permission to participate in the present study. The 14 high schools belonged to different geographical areas of Catalonia and included participants of different socioeconomic statuses to ensure that the results were representative.

The evaluation was carried out during the same academic year. Once the high schools showed interest, our research team went in person to explain the details of the research, resolve doubts, and specify the procedure. All the students from the same high school were evaluated on the same day by a member of the research team, together with a teacher from the high school. Besides supervising the administration of the paper-and-pencil self-administered survey, our research team addressed the potential doubts of the students. There was no financial reward. However, at the end of the sample collection, our research team returned to each high school to explain, to the boards of education, the main results of the research. It is not possible to calculate the refusal rate because some centers chose not to provide us with this information, but we estimate that it was less than 2%.

### 2.2. Assessment

The survey contained 102 items assessing dispositional, developmental, social, criterion, and media use variables. The items included have not been evaluated for their psychometric properties. Due to practical issues of time and adolescent fatigue, we decided to design items to evaluate the variables of interest instead of using validated psychometric instruments, which are much more extensive.

#### 2.2.1. Dispositional Variables

Dispositional variables included: sociodemographic, norm-related, and sexual interest variables—Internet behavior variables. The sociodemographic variables assessed in the survey were gender and sexual orientation. Drug use and religion were evaluated in the category of norm-related features. Frequency of drug use was coded into one of four categories: non-consumption, once a month or less, between twice a month and once a week, and more than once a week.

#### 2.2.2. Developmental Variables

Developmental variables included age and sexual experience. Sexual experience assessed aspects such as the age of their first sexual experience and the current frequency of sexual intercourse. 

#### 2.2.3. Social Variables

Social variables contained family-related factors and victimization. Family-related factors included items related to the adolescent’s nuclear family and the possible presence of siblings. The victimization section evaluated sexual assault, malpractice during sexting, and online victimization.

#### 2.2.4. Criterion Variables

Criterion variables assessed the following domains: risky sexual behaviors (such as unprotected sex, and sex after alcohol and substance use), and permissive sexual attitudes (such as infidelity).

#### 2.2.5. Media Use

Survey items measured pornography use and related sexual behaviors, sexting, and cybersex behaviors with responses coded dichotomously as “yes/no”.

### 2.3. Statistical Analysis

Statistical analysis was carried out with Stata16 for Windows [36]. A logistic regression fitted predictive models of the pornography media use. Different logistic models were fitted for each of the variables defined as dependent variables (downloading sexual content, use of social nets to send sex content, participation in sexual chats and use of erotic lines). The set of the potential predictors included all the other variables analyzed for this work (dispositional variables (sex, sexual orientation, drug use/abuse, brought up following a religion, religious practitioner, feeling religious, interest in social nets for obtaining sexual content), developmental variables (age, age at first sexual experience and frequency of sexual experiences), and social variables (persons living at home, being abused and being forced to share sex content)). A stepwise method was used to build a final model in which the choice and selection of the significant predictors is carried out by an automatic procedure, adding or removing in subsequent steps the predictors according to pre-specified parameters. This method is particularly useful in studies with a large set of potential independent variables and no underlying empirical hypothesis on which to base the model selection. For categorical independent variables, different contrasts were defined: pairwise comparisons for nonordered variables and polynomial contrasts for ordered variables (polynomial post-hoc tests are particularly useful to determine whether a particular mathematical pattern emerges for the levels of the predictor, such as linear, quadratic, cubic or quartic levels) [37]. Adequate goodness of fit for the final models was considered for nonsignificant results (*p* > 0.05) in the Hosmer‒Lemeshow test. Nagelkerke’s R-squared coefficient (N-R^2^) estimated the global predictive capacity, considering null for N-R^2^ < 0.02, low-poor for N-R^2^ > 0.02, mild-moderate for N-R^2^ > 0.13, and high-good for N-R^2^ > 0.26 [38]. The area under the receiver operating characteristic (ROC) curve (AUC) measured the discriminative capacity (AUC < 0.65 was interpreted as low-poor, AUC > 0.65 mild-moderate, and AUC > 0.70 high-good [39]).

Path analysis was used to describe the underlying mechanisms explaining the pornography use based on the set of variables registered in this work. Path analysis procedures represent a straightforward extension of multiple regression modeling, which allows estimating the magnitude and significance level of associations into a set of variables, including mediational links [40]. This procedure can be used for both exploratory and confirmatory modeling, and therefore it allows theory testing and theory development [41,42]. In this work, and due to the existence of multiple criteria measures, we defined a latent variable defined by the observed indicators contraception, unprotected sex, emergency contraception, practicing sex after alcohol use/abuse, practicing sex after drugs use/abuse and infidelity (the latent variable in this study allowed us to simplify the data structure and therefore facilitated a more parsimonious fitting) [43]. In this study, path analysis was adjusted through Structural Equation Modeling (SEM), using the maximum-likelihood estimation for the parameter estimation, and valuing the goodness of fit through the standard statistical measures: the root mean square error of approximation (RMSEA), Bentler’s Comparative Fit Index (CFI), the Tucker‒Lewis Index (TLI), and the standardized root mean square residual (SRMR). An adequate fit was considered for models meting the next criteria Barret [44]: RMSEA < 0.08, TLI > 0.90, CFI > 0.90, and SRMR < 0.10. The global predictive capacity of the model was measured by the coefficient of determination (CD), whose interpretation is similar to global R^2^ in multivariate regression models.

### 2.4. Ethics

The Hospital Ethics Committee (Comité Ético de Investigación Clínica del Grupo Hospitalario Quiron) approved the procedures of this study (REF: 012/107) in December 2014. The present study was carried out in accordance with the latest version of the Declaration of Helsinki. We obtained a permit from the management boards of each school that agreed to participate in our study. Each school provided the parents or legal guardians of underage students with information about the study. Those parents or minors who did not wish to participate informed the school board. It was clarified that participation was voluntary and they could withdraw at any time. The data of *n* = 1 student were withdrawn from the study after the request of the school board.

## 3. Results

### 3.1. Characteristics of the Sample

Table 1 includes the distribution for the variables analyzed in the study. Most individuals reported heterosexual orientation (90.5%), while 2.1% indicated that they were homosexual, 3.9% bisexual, and 3.6% not defined. The percentage of individuals brought up Catholic was 36.1%, Muslim 4.9%, and other religions 5.3% (the remaining 53.8% indicated that they were atheist). Only 10.7% described themselves as a religious practitioner, with 17.0% being religious or very religious. Around 20% of the sample reported substance use or abuse. The percentage of adolescents who reported sexual interest and the use of the media to obtain sexual information was 25.6%.

The proportion of individuals with sexual experience was around 33%, with 15–16 years old being the most likely age of sexual initiation. The prevalence of adolescents who indicated being victims of sexual abuse was 6.5%, while 17.6% indicated that they had been forced to share sexual content.

Regarding media use, 43.6% reported pornography use. Other related behaviors showed lower percentages (between 6.1% for use of erotic telephone lines and 9.5% for downloading sexual content). The criterion variables were distributed as follows: 31.0% used contraception, 17.3% reported unprotected sex, and 8.7% used emergency contraception; sexual behavior after alcohol use was reported by 29.9% of the participants, while sex after substance use was reported by 11.7%. The percentage of adolescents who reported being unfaithful was 15.7%.

### 3.2. Predictive Models of Pornography Use 

Table 2 contains the results of the logistic regression, selecting the best predictors of pornography use in the study. This model achieved adequate fitting (*p* = 0.385 in the Hosmer–Lemeshow test), large predictive capacity (N-R^2^ = 0.32), and large discriminative capacity (AUC = 0.79). Increases in the odds of pornography use were related to being male, older, bisexual or with undefined sexual orientation, higher substance use, and reporting sexual interest and the use of the media to obtain sexual information; in addition, being Muslim (compared to being atheist) decreased the likelihood of pornography use.

Table 3 contains the results of the logistic models obtained for the other predictors of the pornography use and cybersex behaviors analyzed in this work. Downloading sexual content was most probable for males, those with a bisexual orientation, those reporting sexual interest and the use of social networks to obtain information regarding sex and earlier first sexual experiences. The use of social media to send sexual content was more likely for males, those who use drugs, those with sexual interest who use social media to obtain information about sex, and those who had been sexually abused by adults or other adolescents. The use of social media to send sexual content to others was related to bisexual orientation, sexual interest and the use of social media to obtain sexual information, earlier first sexual experiences, being a victim of sexual abuse, and being forced to share sexual content. The odds of participation in sexual chats was higher for males, those with sexual interest, those who use social media to obtain sexual information and those who have been forced to share sexual content. Finally, the use of erotic telephone lines was higher for men, participants with higher substance use, younger respondents, and those with a higher frequency of sexual experiences.

### 3.3. Path Analysis

**Figure 1:**
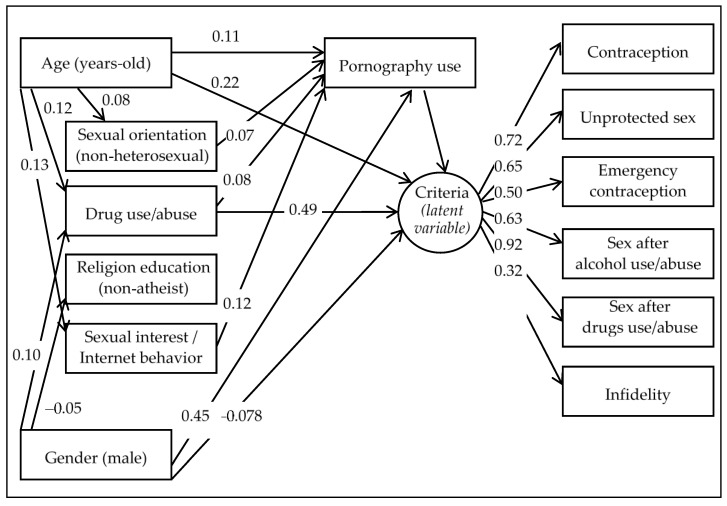
Path diagrams: standardized coefficients in the Structural Equation Modeling (SEM) (*n* = 1500). Note: Only significant parameters were retained in the model.

Figure 1 includes the path diagram with the standardized coefficients obtained in the SEM, in which only significant parameters were retained (only relationships with significance levels *p* < 0.05 are plotted). Figure 1 uses conventional rules for path diagrams and SEM schemes; the observed variables are drawn by rectangular boxes, while the latent variable is represented by a circular/elliptical shape. The final model obtained in this work met the criteria of all goodness-of-fit indexes: RMSEA = 0.062, CFI = 0.922, TLI = 0.901, and SRMR = 0.050. In addition, a large global predictive capacity was obtained for the model (CD = 0.31). 

All the variables used for defining the latent variable in this study (labeled as “criteria” in the path diagram, Figure 1) achieved high and significant coefficients, the highest score being for practicing sex after substance use/abuse (0.92) and the lowest for infidelity (0.32). The positive coefficients achieved in all the variables defining this latent variable indicate that higher scores in the latent class are indicative of a higher number of behaviors related with risky sexual practices (a high level in the latent variable indicates a high likelihood of contraception use, unprotected sex, emergency contraception, sex practices after alcohol use/abuse, sex practices after drugs use/abuse and infidelity).

The higher levels in the criterion are directly related to pornography use, older age, substance use, and being female. Some mediational links also emerged. Firstly, pornography use mediated between age and criterion variables, as well as between sexual orientation, substance use, and sexual interest and the use of media to obtain information regarding sex with criterion variables. Secondly, substance use also mediated in the correlation between age and gender with the criterion variables. Religious education did not achieve direct/indirect contribution on the pornography use and on the latent variable.

## 4. Discussion

The purpose of this research was two-fold: (1) to explore whether dispositional, developmental, and social variables predict pornography use; (2) to evaluate whether these variables not only predict pornography use but also moderate the extent to which pornography use predicts criterion variables.

Regarding dispositional variables, sexual orientation is a relevant multidimensional construct that has been widely evaluated in the adult population [45,46]. However, the prevalence of sexual minority identity has rarely been examined in adolescents [47]. In the present study, 6% of the sample identified as lesbian, gay, or bisexual (LGB) and 3.6% did not define their sexual orientation. These percentages are not far removed from previous studies. For example, Li et al. [48] found that approximately 4% of adolescents self-identified as LGB, while 14% were unsure of their sexual orientation. 

When examining norm-related features, also included in the dispositional variables, religiosity seems to be another factor related to adolescent sexuality [49]. In the present study, the percentage of Catholic adolescents was 36.1%, Muslims was 4.9%, and other religions was 5.3%. Other studies that have evaluated religiosity and sexuality in adolescents have found much higher rates of religiosity. For example, 83% of the adolescents in Mexico report being Catholic [50]. The prevalence is closely linked to the history and culture of each country, making it difficult to generalize. In conjunction, substance use reduces social inhibition and is associated with increased risk-taking behaviors, especially in the area of sexuality [51,52]. In adolescent populations, rates of substance use are very heterogeneous and range from 0.4% to 46% [53,54,55,56]. These results coincide with our findings, given that around 20% of our sample reported substance use or abuse.

Finally, sexual interest has also been considered as a dispositional variable in the present study. The percentage of adolescents who reported sexual interest and who used digital media to obtain sexual information was 25.6%. Studies in this field have detected an increase in searching for information on sex among adolescents since the emergence of the Internet [57]. In addition, there seems to be an association between those adolescents who engage in more risky sexual behaviors and the likelihood of seeking this type of information on the Internet [58]. Some of the barriers that adolescents report when doing this type of search are the overabundant content that is difficult to filter out, as well as complaints about unintentional exposure to sexually explicit content during these searches [59].

With respect to developmental variables, the proportion of individuals in the present study with sexual experience was around 33%, a figure similar to the 28.1% reported in previous studies [60]. Moreover, 15–16 years old was the most frequent age of initiation of sexual behavior in our sample. Other studies in this line have reported ages of sexual initiation around 12.8–14 years old [61]. These differences could be due to multiple causes. As some authors suggested, early sexual initiation may be influenced by factors such as alcohol use, the involvement of chat rooms or dating websites, and the use of medication for mental problems [62,63]. However, although the percentages vary, all comprise early sexual initiation (<16 years old) [64].

Regarding social variables, and victimization more specifically, 6.5% of the adolescents reported being victims of sexual abuse. The rate of sexual abuse or assault in other European countries is about 14.6% [65]. Although it is a more common problem among adolescent females, there is a growing recognition that sexual victimization is also a relevant, though invisible, issue among male adolescents [66,67]. In this line, 17.6% of our sample reported being forced to share sexual content through social media. This pressure and the diffusion of sexual content without consent derived from sexting, as well as other online victimization behaviors such as revenge porn, cyberbullying, and online dating violence, are increasingly present in the adolescent population [68,69]. Titchen et al. [70] observed that more than three times as many girls as boys felt pressured to send a sext. They also found an association between sexual abuse and sexting in both sexes, thus suggesting that sexual abuse may lead to early sexualization.

Finally, with regard to media use, 43.6% of adolescents reported the use of pornography, 9.5% reported downloads of sexually explicit materials, and 6.1% engaged in phone sex. The pornography use prevalence was similar to other studies, which reported it to be around 43% [5]. However, these percentages are much lower than those found by other studies in adolescents and young adults, which ranged from 80% to 96% [71,72,73]. 

As the DSMM suggests [9], dispositional, developmental, and social variables were related to pornography use in our study. More specifically, increases in the odds of pornography use were associated with being male, older, bisexual or with an undefined sexual orientation, substance use, not being Muslim, and higher sexual interest and use of social media to obtain sexual information. These findings are consistent with other studies highlighting that male and female adolescents differ in their patterns of consumption of pornography [74,75]. This could be partially explained by the greater tendency of males to rate sexual stimuli as more pleasant and arousing and to show stronger neural responses derived from exposure to these sexual stimuli [76,77]. However, a slight increase in female pornography use over time has been identified (28% in the 1970s vs. 34% in the 2000s) [78]. Studies exploring the reasons for these sexual differences in pornography use are still very scarce. However, some authors have suggested that some factors may promote female pornography use, such as the rise of feminist porn with less aggressive content, younger age, absence of religiosity, and higher education levels [78,79]. Sexual orientation has also been a factor associated with pornography use. Our findings corroborate previous studies suggesting greater pornography use by bisexual than by heterosexual adolescents [35,80]. However, most studies do not assess sexual orientation or focus only on heterosexual adolescents [14]. Therefore, more research is needed, including with under-represented sexual minorities. A significant association was also found between pornography use and substance use, which is consistent with previous findings [19,81]. Some authors suggest that this correlation may be influenced by factors such as high sensation-seeking levels [81]. Considering the link between religion and pornography use, numerous studies have been based on moral incongruence [82,83]. This addresses the incompatibility between pornography use and an individual’s deeply held values and beliefs about the inappropriateness of that behavior [84]. Pornography use seems to be lower with higher levels of religious attendance, especially among male adolescents, and religious attendance weakens age-based increases in pornography use for both sexes [85]. 

In addition, we studied whether pornography use predicted criterion variables through SEM, as proposed by the DSMM [9]. We observed a direct association between pornography and the following criterion variables: contraception, unprotected sex, emergency contraception, sex after alcohol and other substances, and infidelity. Pornography is associated with a greater tendency to engage in risky sexual behavior, such as sex under the influence of alcohol and other substances, or the use of emergency contraception. These findings corroborate that exposure to pornography may affect psychosexual development in adolescents. More specifically, pornography could lead to more permissive sexual values and changes in sexual behavior, such as an increase in risky sexual behaviors [31,86]. However, these are controversial findings that should be interpreted with caution. Other studies have failed to find an association between exposure to pornography and risky sexual behaviors such as multiple sexual partners, history of pregnancies, or early sexual initiation [35].

### 4.1. Clinical Implications

Although interest in sexuality and pornography use in adolescence has been increasing in recent years, there are still few studies that evaluate the association between these factors and other relevant aspects of this stage of development. It is essential, therefore, to have studies that attempt to design and test theoretical models that allow for the conceptualization and identification of possible phenotypes associated with the use of pornography in adolescents. 

Furthermore, to date, the distance between the research and clinical fields is marked, so an approach is required that favors adequate care for adolescents who demand help for problematic pornography use. 

At the clinical level, it will be of interest to assess the use of pornography in clinical evaluations in order to determine how pornography may be influencing adolescent psychosexual development. In addition, if the person is frequently using pornography, the sexual lifestyle and quality of life, as well as possible sexual risk behaviors, should be taken into account. Problematic pornography use may also be associated with other psychiatric conditions, so detecting them may help to address the consequences of these conditions. In this line, assessing adolescent pornography use can help to detect early maladaptive personality traits, such as high novelty seeking or reward dependence.

An adequate understanding of the interaction between these multiple variables associated with the use of pornography would allow clinical professionals to carry out better prevention, early detection and diagnosis of problems related to adolescent sexuality. Correctly detecting predisposing and precipitating factors of pornography use, as well as possible consequences of pornography use, could also help clinicians to differentiate between pornography use and problematic pornography use, a construct that is becoming increasingly important, both in the clinical setting and in the research field. 

Finally, addressing issues of sexuality in adolescence would reduce the incidence of problems with sexual function and/or hypersexuality in adulthood, the prevalence of which appears to be increasing. 

### 4.2. Limitations 

The results of this study should be considered in light of its limitations. First, the cross-sectional design of the study does not allow for the determination of causal relationships or changes in patterns of adolescent pornography use. Second, the sample is not representative of the entire country, so caution should be exercised when generalizing the results. Third, the survey included many dichotomous items and was not based on validated psychometric questionnaires, which could limit the accuracy of the data obtained. Furthermore, the survey did not provide a specific definition of pornography, which could lead to different interpretations of the term. Fourth, despite the fact that adolescents knew that the evaluation was completely anonymous, when it comes to sexuality we must not forget a possible social desirability bias. Fifth, apart from substance abuse, no common psychopathology was assessed in the adolescent population, such as the presence of behavioral addictions. Finally, the frequency of pornography use was not evaluated, so we were not able to distinguish cases of problematic pornography use.

## 5. Conclusions

Our findings support the clinical applicability of the theoretical DSMM framework. Therefore, dispositional, developmental, and social variables may predict pornography use and may moderate the extent to which pornography uses predicts criterion variables. However, it must be taken into account that not all the variables included in the study had the same relevance in this association. Furthermore, the literature in this field is extremely controversial. Therefore, more studies and a longitudinal design would be necessary to define the profile of adolescent consumers of pornography. Knowing in depth the impact of pornography on this population would also allow for the design of more effective prevention and regulation proposals.

## Figures and Tables

**Table 1:** Descriptive variables of the study (*n* = 1500).

Dispositional Variables		*n*	%	Social Variables		*n*	%
Gender	Female	833	55.5%	Living with …	Both parents	1424	94.9%
	Male	667	44.5%		Other family	40	2.7%
Sexual orientation	Heterosexual	1357	90.5%		Other	36	2.4%
	Homosexual	31	2.1%	Living with …	0 Siblings	350	23.3%
	Bisexual		3.9%		1 sibling	818	54.5%
	Not defined	54	3.6%		2 siblings	229	15.3%
Substance use/abuse	Never	1212	80.8%		3 or more siblings	103	6.9%
	1×/month or less	126	8.4%	Sexual abuse	No	1403	93.5%
	2×/month or 1×/week	75	5.0%		Yes	97	6.5%
	3× or more/month, or weekly	87	5.8%	Forced to share sexual content	No	1236	82.4%
Brought up in a religion	Atheist	807	53.8%		Yes	264	17.6%
	Catholic	541	36.1%	Pornography media use			
	Muslim	73	4.9%	Pornography use	Yes	654	43.6%
	Other	79	5.3%	Social media to send sex content	Yes	112	7.5%
Religious practitioner	No	1339	89.3%	Social media to send self-sex.	Yes	98	6.5%
	Yes	161	10.7%	Participation in sexual chats	Yes	93	6.2%
Religious feeling	None	936	62.4%	Use of erotic telephone lines	Yes	91	6.1%
	A little religious	308	20.5%	Downloading sexual content	Yes	143	9.5%
	Religious	218	14.5%	Criterion variables			
	Very religious	38	2.5%	Use of contraception	Yes	465	31.0%
Sexual interest/Internet behavior	No	1116	74.4%	Unprotected sex	Yes	260	17.3%
	Yes	384	25.6%	Use of emergency contraception	Yes	130	8.7%
Developmental variables				Sex after alcohol	Yes	449	29.9%
Age	14 years	117	7.8%	Sex after other substances	Yes	176	11.7%
	15 years	340	22.7%	Infidelity	Yes	236	15.7%
	16 years	360	24.0%	Infidelity: caresses	Yes	145	9.7%
	17 years	454	30.3%	Infidelity: kisses	Yes	41	2.7%
	18 years	229	15.3%	Infidelity: embraces	Yes	221	14.7%
Age (years old) Mean—SD		16.23	1.18	Infidelity: oral sex	Yes	58	3.9%
First sexual experience at	Never	1008	67.2%	Infidelity: masturbation	Yes	143	9.5%
	Under 13	20	1.3%	Infidelity: penetration	Yes	44	2.9%
	13–14 years	130	8.7%				
	15–16 years	284	18.9%				
	17–18 years	58	3.9%				
Frequency of sexual experience	Never	1012	67.5%				
	Only 1 time	64	4.3%				
	2–5 times	99	6.6%				
	6–10 times	55	3.7%				
	More than 10 times	270	18.0%				

SD: standard deviation.

**Table 2:** Predictive models of pornography use: stepwise logistic regression (*n* = 1500).

Criterion: Pornography Use. Fitting Indexes: H-L(*p*-Value) = 0.385; N-R^2^ = 318; AUC = 0.790 (95%CI: 0.767 to 0.813)							
Predictor	Contrast	B	SE	*p*	OR	95%CI	
Sex	Male vs. Female	2.122	0.128	<0.001	8.349	6.503	10.720
Sexual orientation				0.019			
	Homosex. vs. Heterosex.	0.221	0.425	0.603	1.247	0.543	2.867
	Ambisex. vs. Heterosex.	0.702	0.307	0.022	2.019	1.107	3.682
	Non-defined vs. Heterosex.	0.738	0.324	0.023	2.092	1.108	3.949
Drugs use/abuse				0.003			
	Linear trend	0.413	0.192	0.032	1.511	1.036	2.202
	Quadratic trend	−0.364	0.214	0.088	0.695	0.457	1.056
	Cubic trend	−0.113	0.233	0.627	0.893	0.566	1.410
Brought up in religion				0.064			
	Catholic vs. Atheist	0.028	0.131	0.832	1.028	0.796	1.328
	Muslim vs. Atheist	−0.771	0.300	0.010	0.463	0.257	0.833
	Other vs. Atheist	−0.159	0.274	0.562	0.853	0.498	1.460
Sexual interest/Internet behavior	Yes vs. No	0.747	0.139	<0.001	2.112	1.608	2.773
Age (years-old)		0.252	0.053	<0.001	1.287	1.159	1.429

H-L: Hosmer–Lemeshow. N-R^2^: Nagelkerke’s pseudo-R^2^ coefficient. AUC: Area under the ROC curve. B: Logistic parameter. SE: Standard error. OR: odds ratio.

**Table 3:** Predictive models of pornography use and cybersex behaviors: stepwise logistic regression (*n* = 1500).

Criterion: Downloading Sexually Explicit Material. Fitting Indexes: H‒L (*p*-Value) = 0.193; N-R^2^ = 0.155; AUC = 0.748 (95% CI: 0.709 to 0.787).							
Predictor	Contrast	B	SE	*p*	OR	95%CI	
Sex	Male vs. Female	1.554	0.211	<0.001	4.730	3.126	7.157
Sexual orientation				0.011			
	Homosex.vs. Heterosex.	−0.774	0.761	0.309	0.461	0.104	2.050
	Ambisex. vs. Heterosex.	1.147	0.372	0.002	3.149	1.519	6.530
	Non-defined vs. Heterosex.	−0.293	0.560	0.601	0.746	0.249	2.235
Sexual interest/Internet beh.	Yes vs. No	0.916	0.191	<0.001	2.498	1.718	3.632
1st sexual experience at age…				0.006			
	Linear trend	0.222	0.289	0.442	1.249	0.709	2.200
	Quadratic trend	−0.053	0.297	0.858	0.948	0.530	1.697
	Cubic trend	1.086	0.360	0.003	2.961	1.462	5.997
	Quartic trend	−0.561	0.347	0.106	0.571	0.289	1.126
Criterion: using social media to send sexual content. Fitting indexes: H‒L (*p*-value) = 0.755; N-R^2^ = 0.181; AUC = 0.776 (95% CI: 0.728 to 0.825).							
Predictor		B	SE	*p*	OR	95%CI	
Sex	Male vs. Female	0.989	0.221	<0.001	2.690	1.744	4.149
Drugs use/abuse				0.022			
	Linear trend	0.415	0.260	0.111	1.514	0.909	2.523
	Quadratic trend	0.025	0.320	0.936	1.026	0.548	1.920
	Cubic trend	0.603	0.368	0.101	1.827	0.889	3.755
Sexual interest/Internet behavior	Yes vs. No	1.705	0.210	<0.001	5.504	3.647	8.306
He/she has been abused	Yes vs. No	1.372	0.308	<0.001	3.943	2.156	7.210
Criterion: using social media to send self-sexual material. Fitting indexes: H‒L (*p*-value) = 0.554; N-R^2^ = 0.190; AUC = 0.790 (95% CI: 0.740 to 0.841).							
Predictor		B	SE	*p*	OR	95%CI	
Sexual orientation				0.001			
	Homosex. vs. Heterosex	0.842	0.560	0.133	2.320	0.774	6.960
	Ambisex. vs. Heterosex	1.289	0.360	<0.001	3.630	1.791	7.356
	Non-defined. vs. Heterosex.	0.750	0.464	0.106	2.116	0.853	5.252
Sexual interest/Internet behavior.	Yes vs. No	1.295	0.225	<0.001	3.650	2.349	5.669
1st sexual experience at age…				<0.001			
	Linear trend	0.670	0.325	0.039	1.955	1.034	3.697
	Quadratic trend	−0.120	0.328	0.716	0.887	0.466	1.689
	Cubic trend	1.023	0.404	0.011	2.782	1.261	6.135
	Quartic trend	−0.714	0.374	0.056	0.490	0.235	1.019
He/she has been abused	Yes vs. No	1.021	0.310	0.001	2.776	1.512	5.098
Forced to share sex content	Yes vs. No	0.595	0.247	0.016	1.813	1.117	2.941
Criterion: participation in sexual chats. Fitting indexes: H‒L (*p*-value) = 0.878; N-R^2^ = 0.045; AUC = 0.642 (95% CI: 0.582 to 0.702).							
Predictor		B	SE	*p*	OR	95%CI	
Sex	Male vs. Female	0.684	0.227	0.003	1.983	1.270	3.095
Sexual interest/Internet behavior.	Yes vs. No	0.588	0.224	0.009	1.801	1.161	2.795
Forced to share sex content	Yes vs. No	0.907	0.251	<0.001	2.477	1.515	4.047
Criterion: use of erotic telephone lines. Fitting indexes: H-L (*p*-value) = 0.744; N-R^2^ = 0.083; AUC = 0.703 (95% CI: 0.646 to 0.761).							
Predictor		B	SE	*p*	OR	95%CI	
Sex	Male. vs. Female	0.730	0.231	0.002	2.074	1.319	3.263
Drugs use/abuse				0.019			
	Linear trend	0.566	0.280	0.043	1.762	1.018	3.050
	Quadratic trend	−0.356	0.302	0.238	0.701	0.388	1.265
	Cubic trend	0.147	0.332	0.659	1.158	0.604	2.218
Age (years-old)		−0.234	0.101	0.021	0.791	0.649	0.965
Frequency sexual experiences				0.001			
	Linear trend	−0.215	0.342	0.530	0.807	0.413	1.576
	Quadratic trend	−0.713	0.315	0.024	0.490	0.264	0.909
	Cubic trend	0.784	0.530	0.139	2.190	0.774	6.194
	Quartic trend	0.708	0.445	0.112	2.031	0.849	4.860

H-L: Hosmer‒Lemeshow. N-R^2^: Nagelkerke’s pseudo-R^2^ coefficient. AUC: Area under the ROC curve. B: Logistic parameter. SE: Standard error. OR: odds ratio.
